# Identification of key genes regulating brown adipose tissue thermogenesis in goat kids (*Capra hircus*) by using weighted gene co-expression network analysis

**DOI:** 10.3389/fvets.2025.1525437

**Published:** 2025-05-14

**Authors:** Minhao Li, Qingjun Zhu, Haili Yang, Yunyi Hu, Le Zhao, Yongju Zhao

**Affiliations:** Chongqing Key Laboratory of Herbivore Science, College of Animal Science and Technology, Southwest University, Chongqing, China

**Keywords:** brown adipose tissue, thermogenesis, WGCNA, goat kids, key genes

## Abstract

Brown adipose tissue (BAT) is crucial for the maintenance of body temperature in newborn animals through non-shivering thermogenesis (NST). However, which kind key genes involved in the regulation of BAT thermogenesis and the internal regulation mechanism of heat production in goat BAT were still unclear. In this study, we analyzed the perirenal adipose tissue transcriptome of Dazu black goats from 0, 7, 14, 21 and 28 days after birth using weighted gene co-expression network analysis (WGCNA) to identify key genes involved in the thermogenesis of BAT. Genes were classified into 22 co-expression modules by WGCNA. The turquoise module exhibited high gene expression in D0, with generally lower expression in the later dates. This pattern is consistent with the rapid color, morphological, and thermogenic changes observed in perirenal adipose tissue shortly after birth. GO functional annotation revealed that the genes in the turquoise module were significantly enriched in the mitochondrion, mitochondrial protein-containing complex, cytoplasm, and mitochondrial inner membrane. KEGG pathway enrichment analysis indicated that these genes were predominantly enriched in the signaling pathways of oxidative phosphorylation, thermogenesis, and TCA cycle. By combining the gene co-expression network analysis of the turquoise module genes and the differentially expression genes (DEG) analysis, we identified 5 candidate key genes (*ACO2*, *MRPS27*, *IMMT*, *MRPL12*, and *TUFM*) involved in regulation of perirenal adipose tissue thermogenesis. This finding offer candidate genes that in the regulation of BAT thermogenesis and body temperature maintenance in goat kids.

## 1 Introduction

Adipose tissue is a highly complex and essential energy storage and endocrine organ. It plays an important role in the regulation of a variety of biological processes, including energy metabolism, glucose homeostasis and thermogenesis (1, 2). In mammals, adipose tissue can be divided into white adipose tissue (WAT), brown adipose tissue (BAT), and beige adipose tissue. WAT primarily functions in energy storage and the breakdown of triglycerides into free fatty acids (FFAs) when the body’s energy supply is insufficient. BAT can specifically express high levels of uncoupling protein 1 (UCP1) and contributes to generating heat by mitochondrial uncoupling of oxidative phosphorylation. The energy generated from substrate oxidation is then released as heat instead of being used for ATP synthesis (3, 4).

BAT thermogenesis is crucial for survival and regulated by various factors, including ambient temperature, diet, hormone levels, and metabolites (5–10). Several key genes involved in the regulation of BAT thermogenesis have been identified in mice and other model animals (10–13). BAT also plays a crucial role in newborn and young goats, as studies have shown that the BAT content of lambs peaks at birth and remains at high levels to generate sufficient heat in cold environments (4). The importance of BAT in goats is further evident by the fact that the mortality rate of newborn lambs is significantly increased at colder temperature when coupled with low BAT content (14, 15). However, BAT thermogenesis in lambs appears to be even more critical. The average heat production rate of lambs was found to be higher than that of human infants and piglets under thermoneutral, moderate, and cold conditions (16), indicating the significance of BAT thermogenesis for lambs. Related studies have also identified key regulatory factors and pathways in the regulation of BAT thermogenesis in goats, such as FGF11, LncDGAT2, miR-433, and AMPK pathway (17–20). However, studies on the key genes regulating BAT thermogenesis in goats are still very limited.

In this study, we combined WGCNA and DEG to analyze the transcriptome data of the goat perirenal adipose tissue at different periods (D0, D7, D14, D21, and D28) after birth and to identify the key module and genes regulating BAT thermogenesis. This study provides new knowledge regarding BAT thermogenesis in goats.

## 2 Materials and methods

### 2.1 Sample collection

Dazu black goat, the national livestock and poultry genetic resource, was used as the animal model. A detailed description of the experimental design and sample collection is provided in our previous study (21, 22). A total of 18 perirenal adipose tissue samples from newborn goats were collected at 0 days (*n* = 4), 7 days (*n* = 4), 14 days (*n* = 3), 21 days (*n* = 3), and 28 days (*n* = 4) after birth, then immediately snap frozen in liquid nitrogen and stored at - 80°C until processing and analysis were performed.

### 2.2 Data sources

We obtained 18 previously published RNA-seq datasets (21, 22). In brief, RNA-seq was performed on an Illumina sequencing platform by Genedenovo Biotechnology Co., Ltd. (Guangzhou, China). Quality control and data filtration of raw reads were performed by FASTP software (RRID: SCR_016962) (23). Reads were compared with the reference genome of goats by the HISAT2 software (RRID: SCR_015530) (24). Principal component analysis (PCA) of gene expression data was performed by R^1^ to assess repeatability between samples and exclude outliers.

### 2.3 Weighted gene co-expression network analysis

The data was analyzed using weighted gene co-expression network analysis (WGCNA). Co-expression networks were constructed using WGCNA (v1.47) package in R. After filtering genes, gene expression values were imported into WGCNA to construct co-expression modules using the automatic network construction function block wise Modules with default settings, except that the power was set at 8, TOMType was unsigned, merge Cut Height was 0.85, and min Module Size was 50. Genes were clustered into 22 correlated modules. Key modules related to perirenal adipose tissue thermogenesis were selected by the expression pattern of each module. Intramodular connectivity (K.in) and module correlation degree (MM) of each gene were calculated using the WGCNA R package.

### 2.4 Differentially expression genes analysis

Differences in gene expression levels of perirenal adipose tissues at D0 vs. D7, D0 vs. D14, D0 vs. D21, and D0 vs. D28 were analyzed using the DESeq2 software (RRID: SCR_015687). Differentially expressed mRNAs with a false discovery rate (FDR) < 0.05 and |log2 FC| > 1 were considered significant.

### 2.5 Gene function analysis of the key module

GO (Gene Ontology) functional annotation and KEGG (Kyoto Encyclopedia of Genes and Genomes) pathway enrichment analysis of key module genes was performed using the OmicShare tool (RRID: SCR_025711). The FDR-corrected *p*-value was < 0.05. The module node file and the module edge file (weight > 0.28) were imported into Cytoscape 3.7.2 (RRID: SCR_003032) for network visualization.

### 2.6 RNA extraction, cDNA synthesis, and RT-qPCR

Total RNA of perirenal adipose tissue was extracted using RNAiso Plus Reagent (Takara, Code No.9109, Shiga, Japan), according to the manufacturer’s instructions. The cDNA synthesis was performed using the All-In-One 5 × RT MasterMix Kit (Abm, Cat #G592, Richmond, Canada). RT-qPCR was performed using the Blastaq™ 2 × qPCR MasterMix Kit (Abm, Cat #G891, Richmond, Canada) on a quantitative real-time PCR instrument (Bio-Rad, California, USA). The primers used in this study were designed by Primer-BLAST (NCBI) and are shown in Supplementary Table 3. The 2^-ΔΔCT^ method was used to calculate the relative mRNA expression level.

### 2.7 Statistical analysis

IBM SPSS Statistics 27 (RRID: SCR_016479) and GraphPad Prism 9.5 software (RRID: SCR_002798) were used for data analysis and graph plotting. One-way analysis of variance (ANOVA) was used for multiple comparisons. A *p*-value less than 0.05 was considered statistically significant.

## 3 Results

### 3.1 Weighted gene co-expression network construction and module division

**Figure 1:**
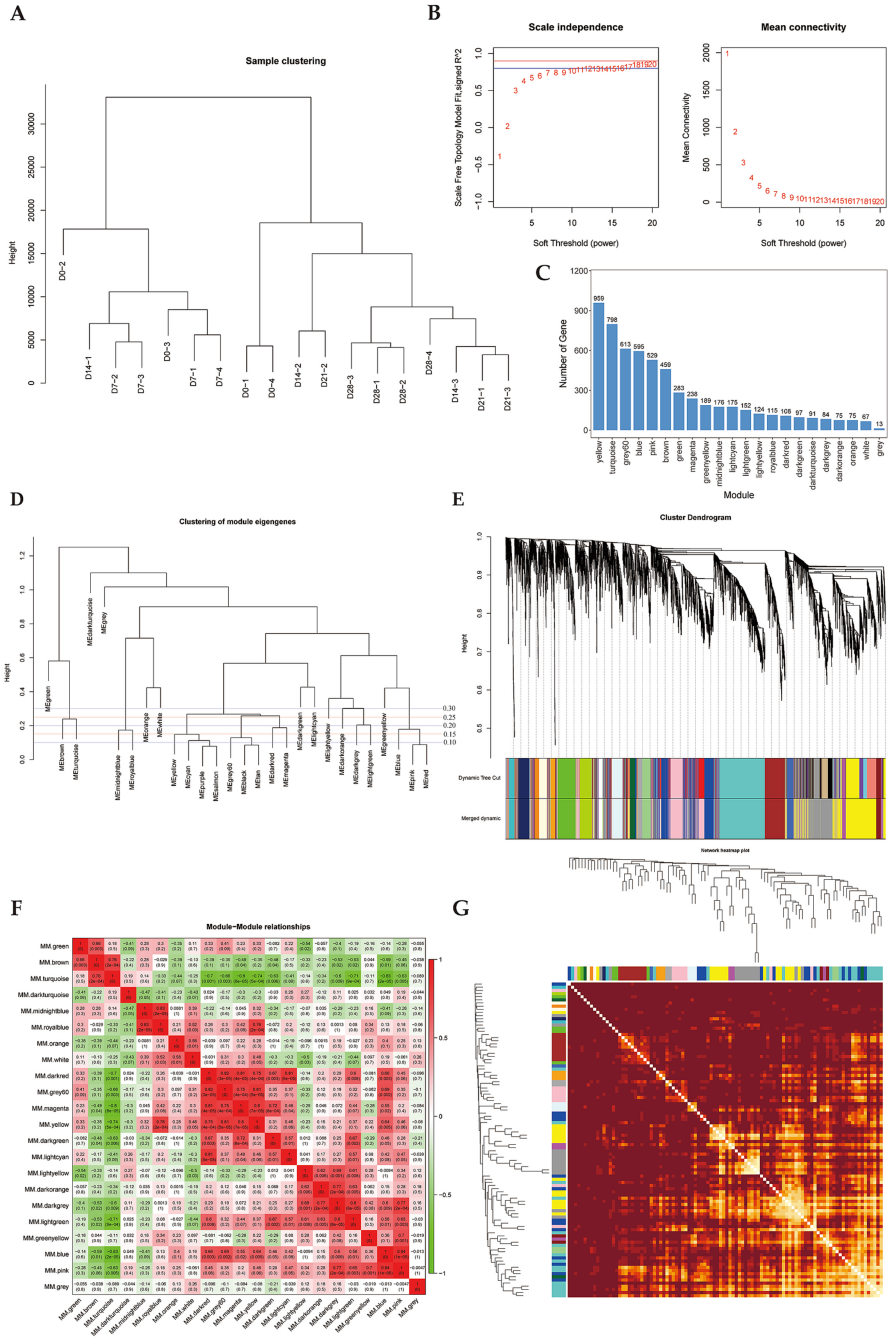
Gene co-expression Module division by WGCNA. **(A)** Hierarchical clustering tree of samples. **(B)** Determination of soft thresholding power, blue line is *R*^2^ = 0.8, red line is *R*^2^ = 0.9. **(C)** Histogram of gene numbers of each module. **(D)** Dendrogram of module eigengenes clustering. Height refers to the uncorrelation between modules, correlation (mergeCutHeight) = 1-uncorrelation. **(E)** Dendrogram of module hierarchic clustering. **(F)** Heatmap of correlation between modules. **(G)** Heatmap of module-gene correlation. The intersection of each branch represents a gene, and the darker the dot color, the stronger the connectivity between two genes corresponding to the row and column.

To investigate key genes regulating perirenal adipose tissue thermogenesis in newborn goats, we analyzed 18 samples from Dazu black goats at 0, 7, 14, 21, and 28 d after birth. A total of 6,015 transcripts were detected using RNA-seq, followed by the construction of a weighted gene co-expression network. Hierarchical clustering of all samples was performed according to the expression level of the genes, and no abnormal samples were detected (Figure 1A). Then, the optimal soft-thresholding power *β* was determined by scale-free topology fit index analysis. The results showed that when *β* = 8, the scale-free fit index (signed *R*^2^) approached 0.8 and reached the plateau, indicating that the network approached the scale-free distribution (Figure 1B). In addition, the mean connectivity was close to 0 when *β* = 8 (Figure 1B), which was also consistent with the characteristics of scale-free networks. Therefore, the optimal soft-thresholding power *β* = 8 was determined. We constructed the gene clustering tree based on the correlation of gene expression levels and divided genes into different modules according to the clustering relationship across genes to generate the Dynamic Tree Cut (Figure 1D). Optimizing the module merging thresholds (mergeCutHeight), and the results showed that when mergeCutHeight = 0.85, the number of merged modules was 22 and the number of genes in each module exceeded 50 except gray module (genes that cannot be grouped into any module) (Figures 1D,E). Among these modules, the yellow module contained the highest number of genes (959 genes), while the gray module had the fewest (13 genes) (Figure 1C). To analyze the connectivity relationships across modules, we generated the heatmap of module correlations (Figure 1F), and generated the heatmap of module genes correlation based on the correlation between gene expression and module feature values (Figure 1G).

### 3.2 Identification of key modules related to the thermogenesis of perirenal adipose tissue

**Figure 2:**
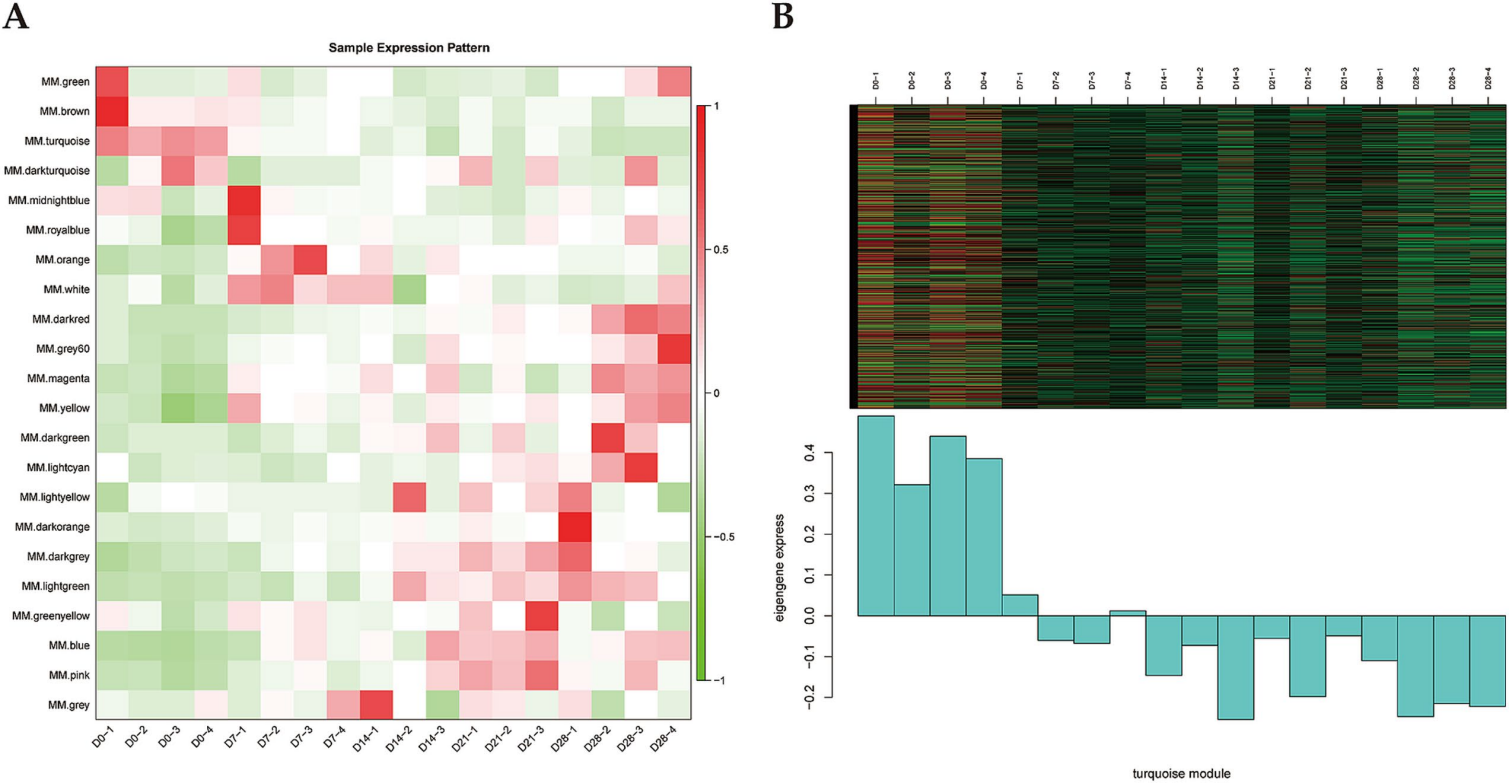
Identification of key module. **(A)** Heatmap of sample expression pattern (x-axis is the sample and y-axis is the module). Red represents high expression and green represents low expression. **(B)** Heatmap and histogram of gene expression pattern of the turquoise module.

Gene expression patterns of each module was analyzed using module feature values, and then the heatmap of the module gene expression patterns was generated (Figure 2A). We found that the white module genes were highly expressed in D7 samples but exhibited lower expression levels in the other samples. The pink, blue, and dark gray modules showed similar expression patterns, characterized by high expression levels in D21 samples. Notably, the turquoise module showed high expression levels in D0 samples but decreased expression levels in the D7, D14, D21, and D28 samples (Figure 2B). Previous studies have demonstrated that BAT is abundant in newborn lambs, particularly in the perirenal regions, then gradually changes to the white-like adipose tissue, with diminished thermogenic activity (25). Interestingly, the expression pattern of the turquoise module corresponded well with this observation. Therefore, we suggested that the turquoise module was the key module related to the thermogenesis of perirenal adipose tissue in newborn goats.

### 3.3 Gene functional enrichment analysis of key module

**Figure 3:**
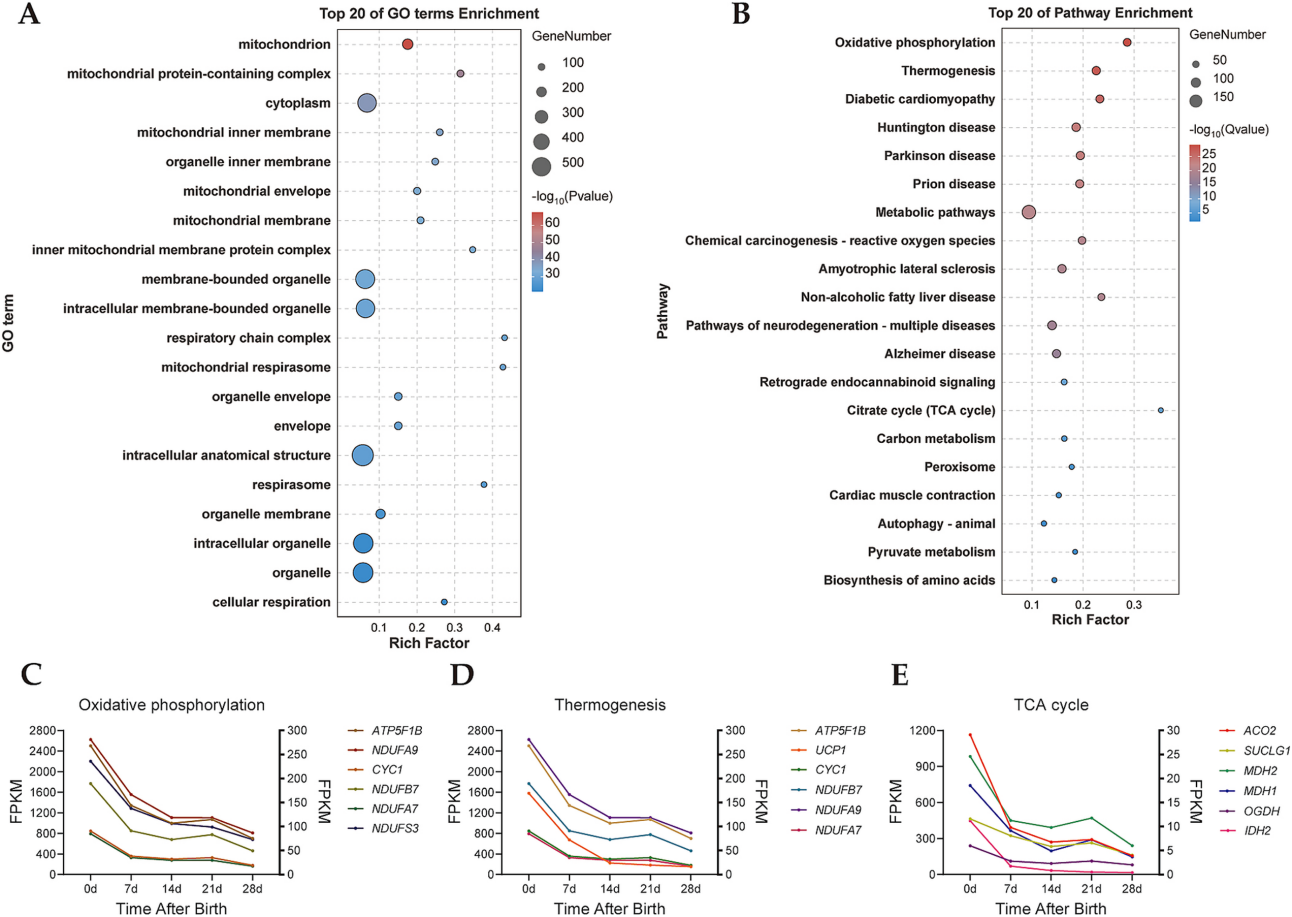
GO and KEGG enrichment analysis of turquoise module. **(A)** GO enrichment analysis of the turquoise module. **(B)** KEGG enrichment analysis of the turquoise module. **(C)** Expression trend of genes in the oxidative phosphorylation. Left ordinate: *ATP5F1B* and *CYC1*, right ordinate: *NDUFA9*, *NDUFB7*, *NDUFA7*, and *NDUFS3*. **(D)** Expression trend of genes in thermogenesis. Left ordinate: *ATP5F1B*, *UCP1* and *CYC1*, right ordinate: *NDUFA9*, *NDUFB7*, and *NDUFA7*. **(E)** Expression trend of genes in the TCA cycle. Left ordinate: *ACO2*, *SUCLG1*, *MDH1*, *OGDH*, and *IDH2*, right ordinate: *MDH2*.

GO and KEGG enrichment analyses of turquoise module genes were performed. The results of GO functional enrichment analysis revealed that the turquoise module genes were significantly enriched in 1,141 GO terms, including annotations related to mitochondria, mitochondrial protein-containing complex, mitochondrial inner membrane, and others (Figure 3A). In the KEGG pathway enrichment analysis, 26 signaling pathways were enriched in the turquoise module (Figure 3B). Notably, pathways such as oxidative phosphorylation, thermogenesis, and the citrate cycle (TCA cycle), which all play significant roles in BAT thermogenesis. Furthermore, we found that genes with high connectivity in the oxidative phosphorylation signaling pathway such as *ATP5F1B*, *CYC1*, *NDUFA9*, *NDUFB7*, *NDUFA7*, and *NDUFS3* all showed high expression in D0 samples (Figure 3C), and genes with high connectivity in the TCA cycle such as *ACO2*, *SUCLG1*, *MDH1*, *OGDH*, and *IDH2* also exhibited a decreasing trend from D0 to D28 samples (Figure 3E). Classical key genes of BAT thermogenesis, *UCP1*, was also highly expressed in D0 samples (Figure 3D). These results further indicated that the turquoise module was the key module involved in regulating thermogenesis of perirenal adipose tissue.

### 3.4 Combined analysis of DEG and co-expression network in key module genes

**Figure 4:**
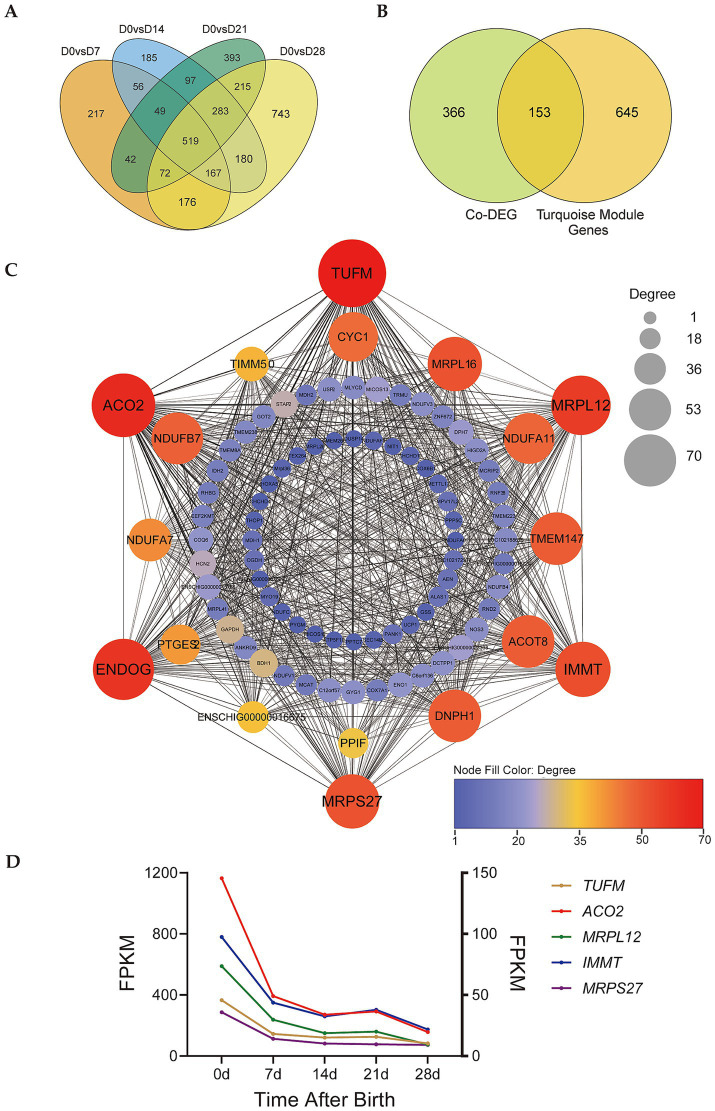
Identification of key genes involved in regulating perirenal adipose tissue thermogenesis. **(A)** Venn diagram of DEGs of D0 vs. D7, D0 vs. D14, D0 vs. D21, and D0 vs. D28. **(B)** Venn diagram of co-DEGs and turquoise module. **(C)** Gene co-expression network of co-DEGs in the turquoise module. Degree indicates node connectivity. **(D)** Expression trend of candidate key genes. Left ordinate: *TUFM* and *ACO2*, right ordinate: *MRPL12*, *IMMT*, and *MRPS27*.

By counting the differentially expressed genes (DEGs) of D0 vs. D7, D0 vs. D14, D0 vs. D21, and D0 vs. D28, we obtained a total of 519 co-differentially expressed genes (co-DEGs) in these 4 groups (Figure 4A, Supplementary Table 1). The 519 co-DEGs were compared to the turquoise module genes to obtain 153 co-DEGs belonging to the turquoise module (Figure 4B, Supplementary Table 2). We constructed the gene co-expression network map of these 153 co-DEGs, setting weight > 0.28 as a screening condition (Figure 4C). Five genes with the highest connectivity (*TUFM*, *ACO2*, *MRPL12*, *IMMT*, and *MRPS27*) were selected for further analysis. All these five genes are localized in the mitochondrion. The *MRPL12* and *MRPS27* genes participate in the formation of mitochondrial ribosomal subunits, the *IMMT* gene participates in the formation of the inner mitochondrial membrane. The *ACO2* gene participates in the TCA cycle, and the *TUFM* gene plays an important role in the mitochondrial protein synthesis. RNA-seq results also showed that these genes were highly expressed in D0 samples, and showed decreasing trends from D0-D28 (Figure 4D).

### 3.5 Validation of relative mRNA expression of candidate genes by RT-qPCR

**Figure 5:**
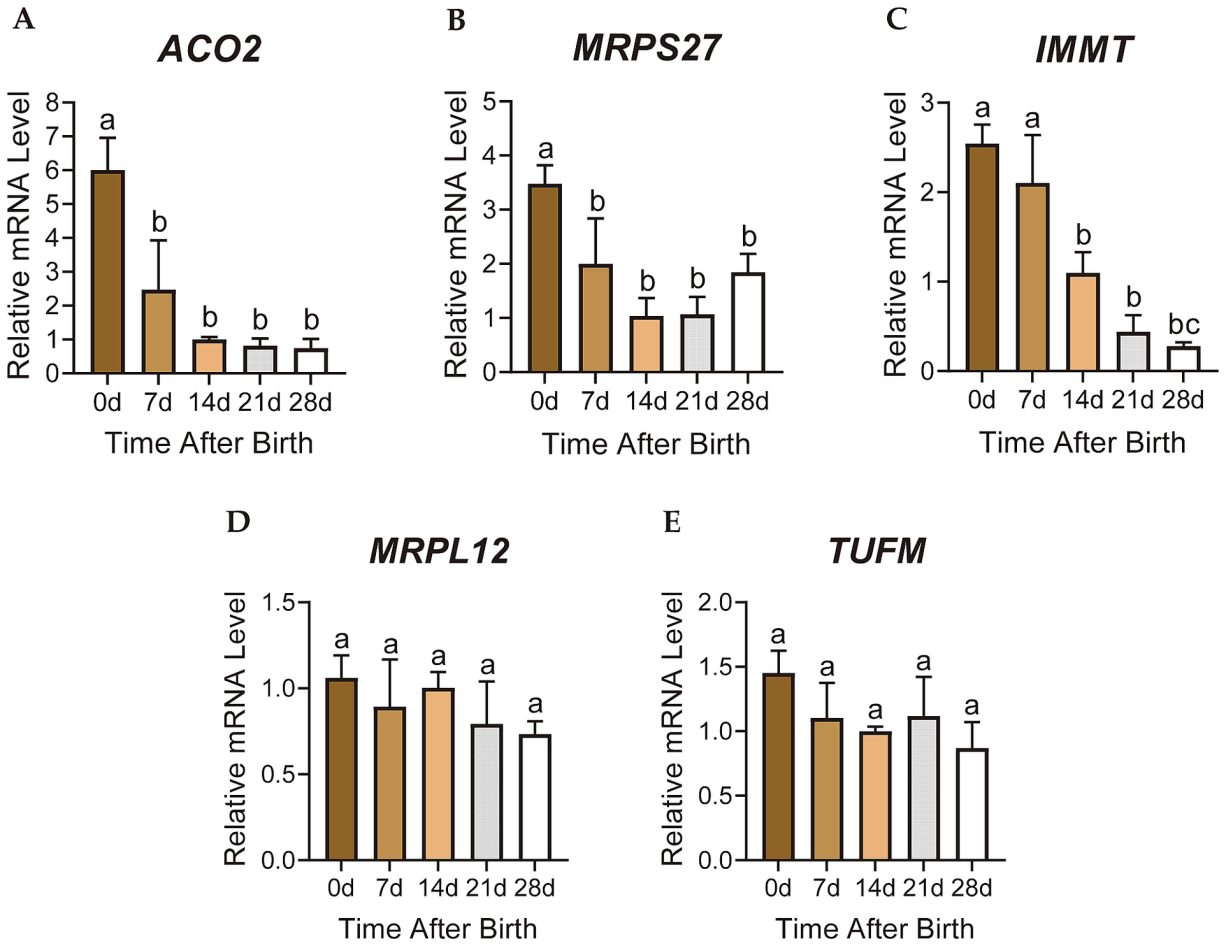
Gene expression analysis of candidate key genes. **(A-E)** The relative mRNA expression of candidate key genes. Different letters represent significance, while the same letters show no significance. The error bar represents Standard Deviation (SD).

The relative mRNA expression levels of the candidate genes *ACO2*, *MRPS27*, *IMMT*, *MRPL12* and *TUFM* were detected by RT-qPCR. The results were consistent with the gene expression patterns of the turquoise module, showing decreasing trends from 0 to 28 days after birth (Figures 5A–E). The expression levels of *ACO2* and *MRPS27* genes in the perirenal adipose tissue of D0 were significantly higher than D7 (*p* < 0.05), D14 (*p* < 0.01), D21 (*p* < 0.01), and D28 (*p* < 0.05) (Figures 5A,B). Although the expression of *IMMT* gene was not significantly different between the D0 and D7 perirenal adipose tissues (*p* > 0.05), it was significantly higher than D14 (*p* < 0.01), D21 (*p* < 0.01), and D28 (*p* < 0.01) (Figure 5C). Both *MRPL12* and *TUFM* genes exhibited the highest expression levels in D0 perirenal adipose tissues, which then decreased over time (Figures 5D,E). The relative mRNA expression levels of these genes in perirenal adipose tissue of newborn goats all showed decreasing trends from 0 to 28 days after birth, which is highly correlated with the change of perirenal adipose tissue from brown to white-like adipose tissue in newborn goats (21), indicating a close correlation with the thermogenesis of perirenal BAT.

## 4 Discussion

The cold environmental represents the first challenge for newborn animals. Understanding the mechanisms of how young animals rapidly adapt to the external environment after birth is critical for improving their survival. BAT is mainly present in high amounts in newborn mammals, due to its great significance in the body temperature maintenance and regulation of newborn animals through non-shivering thermogenesis. In this study, we employed WGCNA to analyze the gene expression levels of perirenal adipose tissues of goats at 0, 7, 14, 21, and 28 days after birth. The relevant genes were divided into 22 co-expression modules. In particular, the turquoise module was highly expressed in D0 samples, and gradually decreased from D0 to D28, which is consistent with the thermogenic changes of goat perirenal adipose tissue in the short term after birth as found in our previous study (21). Therefore, we considered the turquoise module as the key module regulating BAT thermogenesis in goats. The GO and KEGG enrichment analyses of the turquoise module genes showed that they were closely related to mitochondrial components, and were significantly enriched in signaling pathways such as oxidative phosphorylation, thermogenesis, and TCA cycle.

BAT exerts the thermogenic function through the uncoupling function of UCP1, releasing the energy generated by oxidative phosphorylation in the form of heat, while the TCA cycle provides substrates for oxidative phosphorylation (26–28). Oxidative phosphorylation and the TCA cycle both play crucial roles in adipose tissue thermogenesis. Both biological processes are closely related to the metabolic and thermogenic regulation of adipose tissue. Studies have shown that the high activity of the mitochondrial respiratory chain can enhance energy production through oxidative phosphorylation, thereby promoting the thermogenesis of adipose tissue (29, 30). In addition, some enzymes and intermediates in the TCA cycle, such as isocitrate dehydrogenase and succinate, have also been linked to contribute to BAT thermogenesis (31–34). In this study, we also found that the turquoise module contained many genes related to the mitochondrial respiratory chain complexes and TCA cycle, and were highly expressed in D0 perirenal adipose tissue. This link is further evident with the high connectivity of genes involved in BAT thermogenesis (*UCP1* and *PPARGC1a*), oxidative phosphorylation and TCA cycle in the turquoise module. These genes were found to be involved in the metabolic regulation of adipose tissue, including *NDUFA9*, *NDUFS3*, and *IDH2* (31, 35–37). These observations confirm that the turquoise module is indeed the key module regulating BAT thermogenesis. By combining the turquoise module genes with the co-differentially expressed genes of D0 vs. D7, D0 vs. D14, D0 vs. D21 and D0 vs. D28, we were successful in determining the potential key genes (*ACO2*, *MRPS27*, *IMMT*, *MRPLP12*, and *TUFM*) involved in the regulation of the BAT thermogenesis in goats. Remarkably, we found that all the candidate genes are either localized in the mitochondrion, contribute to mitochondrial structure, or participate in mitochondrial metabolism, which further confirms the significance of the mitochondria in BAT thermogenesis. The *ACO2* gene encodes aconitase 2, which is the enzyme that catalyzes the interconversion between citric acid and isocitric acid via cis-aconitic acid through the TCA cycle. Aconitase has two isoforms *in vivo*, ACO1 and ACO2. ACO1 locates in cytoplasm, and studies showed that it was essential for adipocyte differentiation. The expression level of *ACO1* increased synchronously with the differentiation of preadipocytes. Knocking down of *ACO1* impaired the adipogenic differentiation of 3 T3-L1 cells severely (38). *ACO2* can also participate in the metabolic regulation of adipocytes (39). The overexpression of *ACO2* can enhance the differentiation and adipogenesis of 3 T3-L1 cells, thus promoting ATP synthesis (40). In addition, *ACO2* significantly affected mitochondrial functions of adipocytes. The overexpression of *ACO2* promoted mitochondrial biogenesis of 3 T3-L1 cells, while knockdown of *ACO2* significantly inhibited it (41). These results suggested that *ACO2* can regulate adipogenic differentiation and mitochondrial functions of adipocytes, thus may also play a potential role in regulating adipocyte thermogenesis. In this study, we found that *ACO2* is highly expressed in the D0 perirenal adipose tissue of goats. Given its function in adipocytes, it is further confirmed that *ACO2* is a key candidate gene regulating BAT thermogenesis in goats. The *MRPS27* and *MRPL12* genes encode mitochondrial ribosomal proteins S27 and L12, respectively, which are integral components of the mitochondrial ribosomal subunits, and in turn in mitochondrial respiration. The mitochondrial respiratory chain complexes control ATP synthesis and oxidative phosphorylation, and thus can influence thermogenesis of adipose tissues. Knockdown of *MRPS27* can decrease the overall abundance of mitochondrial respiratory complexes (42), which is consistent with our findings that the expression of *MRPS27* in the D0 perirenal adipose tissue of goats was significantly higher than that in the later dates. This suggests that *MRPS27* is highly enriched in the perirenal adipose tissue of newborn goats and could enhance the thermogenesis of adipose tissues by up-regulating the expression of mitochondrial respiratory chain complexes. *MRPL12* is also involved in the regulation of mitochondrial respiration and oxidative phosphorylation, and is associated with long-term high glucose-induced mitochondrial dysfunction (43–45). The expression of *MRPL12* in D0-D28 perirenal adipose tissue of goats showed a decreasing trend, suggesting that *MRPL12* could be involved in the regulation of thermogenesis by influencing the oxidative phosphorylation of adipose tissue mitochondria. The *IMMT* gene encodes the inner membrane mitochondrial protein MIC60, which, along with MIC19 and MIC25, forms the MIC60 subcomplex of the mitochondrial contact site and cristae organizing system (MICOS), which is crucial for the formation of mitochondrial cristae (46, 47). Mitochondrial cristae can effectively increase the surface area of the inner mitochondrial membrane. During the process of adipose tissue thermogenesis, mitochondrial cristae can form the parallel and densely packed sheet network to promote thermogenesis (48–50). Related studies have demonstrated that the protein level of MIC19 in interscapular brown adipose tissue (iBAT) of mice significantly increased in cold conditions and promoted mitochondrial cristae formation, and consequently, thermogenesis (51). Additionally, numerous studies have shown that *IMMT* was essential for the stability of cell mitochondrial cristae structure and MICOS system. Knockout of *IMMT* led to lethal disruption of MICOS complex (52), and knockout of MIC60 resulted in the near-complete loss of protein expression of other MICOS components and severely impaired mitochondrial network structure (53), which emphasizes the importance of *IMMT* and its encoded protein in the formation of mitochondrial network structure and thermogenesis. Studies in adipocytes showed that *miR-378a-3p* directly targeted *IMMT*, regulated mitochondrial functions and metabolism of adipocytes through *circZFYVE9*/*miR-378a-3p*/*IMMT* axis. Overexpression of *miR-378a-3p* significantly inhibited *IMMT* expression, which led to mitochondrial dysfunction and increased oxidative stress of 3 T3-L1 cells (54). In this study, we found that *IMMT* was highly expressed in D0 perirenal adipose tissue of goats, and gradually decreased after D14. Based on the aforementioned studies, we hypothesized that *IMMT* is highly enriched in the BAT of newborn goats to promote the formation of mitochondrial network structures and ensure the thermogenesis of BAT by maintaining the stability of the network structure. The *TUFM* gene encodes the mitochondrial Tu translation elongation factor, a nuclear-encoded mitochondrial protein that forms a complex with mitochondrial GTP and aminoacyl-tRNAs to transport amino acids to the ribosome, and thus plays a key role in mitochondrial proteins synthesis (55, 56). *TUFM* can affect mitochondrial oxidative phosphorylation (57–60), thereby playing a potential regulatory role in energy metabolism and thermogenesis of adipose tissues. Studies have shown that following *TUFM* knockout, the translations of 13 proteins encoded by mitochondrial DNA (mtDNA) were inhibited, all of which are important components of the mitochondrial respiratory chain complex subunits (61). In addition, the expression of *TUFM* was shown to be significantly increased in the adipose tissues of obese human subjects, suggesting its key role in adipose tissue metabolism (62, 63). Related studies have shown that kaempferide (Kaem), a natural compound, acts as an autophagy enhancer to promote autophagy in adipocytes, thus accelerates lipid degradation, and increases *UCP1* expression and thermogenesis of 3 T3-L1 cells. Further studies revealed that Kaem directly targeted *TUFM*, and promoted the interaction between *TUFM* and ATG12-ATG5 complex, thus exerting the regulatory functions on cell autophagy, lipid metabolism and thermogenesis (64). In this study, *TUFM* was highly expressed in D0 perirenal adipose tissue of goats, and its expression showed a decreasing trend over time. Combined with its function in mitochondrial oxidative phosphorylation, we speculated that *TUFM* can promote the synthesis of mitochondrial respiratory chain complexes, and consequently enhance mitochondrial oxidative phosphorylation and BAT thermogenesis.

In summary, we found that *ACO2*, *MRPS27* and *IMMT* genes were differentially expressed in the perirenal adipose tissue of goats at different stages after birth, while the expression of *MRPL12* and *TUFM* gradually decreased over time. These genes played key roles in mitochondrial functions such as mitochondrial biogenesis, mitochondrial respiratory chain complexes assembly, mitochondrial crista structure, mitochondrial protein synthesis, and also affected cell adipogenic differentiation, lipid metabolism, autophagy. Combined with related studies, we hypothesized that these genes coordinate and jointly regulate BAT thermogenesis. Among them, *ACO2* regulated BAT energy metabolism and mitochondrial biogenesis by affecting the progress of key reactions or the production of key substrates of TCA cycle, *MRPL12* and *MRPL27* affected mitochondrial respiration by regulating mitochondrial respiratory chain complex es assembly, *IMMT* maintained the stability of mitochondrial crista structure of brown adipocytes to ensure BAT thermogenesis, *TUFM* regulated the expression of thermogenesis key genes by affecting mitochondrial oxidative phosphorylation or cell autophagy. These candidate genes played a synergistic role in regulating BAT thermogensis from cell energy metabolism, mitochondrial structure and function, oxidative phosphorylation and autophagy. However, although these key candidate genes may play important roles in BAT thermogenesis regulation, there is still a lack of specific functional studies on these genes, and their coordinated regulatory mechanisms need to be further studied in the future.

## 5 Conclusion

In this study, we discovered 22 co-expression modules by WGCNA and identified the key module related to BAT thermogenesis. Using gene co-expression network analysis of the key module and the DEG analysis, we finally obtained 5 key genes (*ACO2*, *MRPS27*, *IMMT*, *MRPL12*, and *TUFM*) that are involved in the regulation of BAT thermogenesis, and speculated that these genes may co-regulate BAT thermogenesis of goat kids by affecting energy metabolism, mitochondrial structure and function, oxidative phosphorylation and cell autophagy. These findings provide new insights for future research in BAT thermogenesis regulation and body temperature maintenance of newborn animals.

## Data Availability

The authors selected the following statement: the datasets presented in this study can be found in online repositories. The names of the repository/repositories and accession number(s) can be found at: https://ngdc.cncb.ac.cn/gsa/, CRA008594.

## Footnotes

^1^ http://www.r-project.org

## Supplementary material

SUPPLEMENTARY TABLE S1 Information of co-differentially expressed genes of D0 vs. D7, D0 vs. D14, D0 vs. D21, and D0 vs. D28.

SUPPLEMENTARY TABLE S2 Information of co-differentially expressed genes belonging to the turquoise module.

SUPPLEMENTARY TABLE S3 Primers used for RT-qPCR.
